# Dysphagia Management in Children: Implementation and Perspectives of Flexible Endoscopic Evaluation of Swallowing (FEES)

**DOI:** 10.3390/children9121857

**Published:** 2022-11-29

**Authors:** Athanasia Printza, Katerina Sdravou, Stefanos Triaridis

**Affiliations:** First Otolaryngology Department, School of Medicine, Aristotle University of Thessaloniki, 54124 Thessaloniki, Greece

**Keywords:** dysphagia, children, flexible endoscopic evaluation of swallowing, FEES, pediatric swallowing disorders, deglutition, feeding

## Abstract

Dysphagia is any impairment of swallowing that compromises the safety, efficiency, or adequacy of nutritional or liquid intake. It is common in children, especially in some clinical populations, and may result in failure to thrive and respiratory problems due to pulmonary aspiration. Swallowing disorders have a severe impact on children’s health, growth, and development, and on the quality of life of the child and family. Clinical evaluation cannot validly predict aspiration, which is mostly silent. A team management approach is advocated, including instrumental swallowing assessments. FEES has been proven to be safe and valid and is increasingly used in children of all ages. It allows the identification of structural abnormalities, assessment of the child’s diet with real-life food and liquids while the child holds the optimal or preferred position, examination during breastfeeding, and assessment of fatigue and treatment strategies. FEES is carried out following a protocol that comprises three parts: the evaluation of the anatomical and physiological parameters of swallowing, testing of food and liquids of a range of different consistencies, and evaluation of treatment methods. Pediatric FEES involves adaptations for infants, and special considerations about readiness for nutritive trials and the infant’s ability to sustain a coordinated feeding pattern. Varying consistencies and volumes of food or liquids are tried. Care of the dysphagic child involves team work. FEES, as a part of the assessment and management of dysphagia, enables the evaluation of the safety, efficiency, and adequacy of oral food and liquid intake. Future perspectives include standardized training in clinical FEES protocols to ensure clinical competency of the pediatric FESS team members and the development and validation of standardized examination and interpretation protocols for pediatric FEES.

## 1. Introduction

Dysphagia is an impairment of the oral intake or food and liquid transportation from the mouth to the stomach. Oropharyngeal swallowing disorders result from impaired oral and pharyngeal phases of swallowing. Esophageal swallowing disorders are related to a disturbed esophageal phase. Depending on the etiology, more than one swallowing phase may be impaired, and oropharyngeal and esophageal dysphagia may coexist or interfere with each other. Swallowing is different in children compared with adults and undergoes developmental changes in the first years of life. Pediatric dysphagia may be congenital or acquired. Feeding difficulties and dysphagia are common in children [1]. They may result in respiratory problems due to pulmonary aspiration and failure to thrive [2]. Dysphagia is any impairment of swallowing that compromises the efficiency, safety, or adequacy of nutritional or liquid intake. As breathing and swallowing are interlinked, a disturbance in either of these processes or failure to synchronize impairs the ability of the child to protect its airway during swallowing. Therefore, eating food and drinking fluids are not safe. Swallowing disorders have a severe impact on children’s health, development, and growth, and on the quality of life of the child and family [3].

In the general population, approximately 1% of children present swallowing difficulties [1]. In some clinical populations, the incidence of dysphagia and feeding difficulties is higher: children with neurological disorders, cerebral palsy, respiratory and cardiac disorders, traumatic brain injury, gastrointestinal disorders, airway malformations, and congenital abnormalities [2]. An increasing prevalence of dysphagia is expected due to the improved survival of very premature children and of children with multimorbidities and severe or complex health issues that are related to swallowing disorders [2,4]. Due to the dysphagia-related patient and family burden, a prompt, valid diagnosis and appropriate management are mandatory. Clinical swallowing evaluations cannot validly predict aspiration in children, and no clinical markers have been proven valid for detecting aspiration in children [5]. Silent aspiration is very common [2]. Valid and reliable diagnosis of dysphagia requires a multidisciplinary team management approach, including instrumental swallowing assessments [6]. In children, the most commonly used instrumental swallowing assessments are videofluoroscopic study of swallowing (VFSS) and flexible endoscopic evaluation of swallowing (FEES) [7]. FEES is increasingly utilized [8]. It has been proven to be a feasible and safe examination procedure in children of all ages [7,8,9]. In a recent historical review of FEES, Langmore described the main benefits of FEES in children as structural abnormalities identification, the ability to examine the child’s diet with liquid and food instead of barium boluses while the child holds the optimal or preferred body position, and the chance to examine swallowing during breastfeeding [8]. Other advantages of FEES are the potential to examine the patient in bed without the need to transport a vulnerable patient to a radiology suite, no need for exposure to radiation, and the direct visualization of the structures and the biomechanics of swallowing. The swallowing evaluation can evolve over a long examination time, allowing assessment of the results of a range of treatment strategies and the impact of fatigue on swallowing [10]. While FEES is increasingly recognized as a useful and safe diagnostic tool in pediatric dysphagia, several issues regarding procedure implementation, protocols, and reporting of outcomes need further investigation and/or standardization.

## 2. Protocols

FEES is a comprehensive examination of swallowing, not a screening exam, to identify aspiration. According to Langmore, FEES is a standardized procedure following a protocol that comprises three parts. The first component is a physiological and anatomical evaluation. The second is the assessment of swallowing of liquids and foods of various consistencies for a range of volume of boluses and varying rates of intake. The third is the evaluation of treatment methods, including positioning, postural maneuvers, effortful swallow, and a wide range of adaptive or compensatory treatments [8,11]. Accumulated oropharyngeal secretions are rated according to the secretion severity scale [12]; airway invasion above or below the level of the vocal folds is rated according to the penetration–aspiration scale [13]; the presence of food or liquid residue in the pharynx after the swallow can be rated with the Yale pharyngeal residue severity rating scale [14]. Pediatric FEES protocols have been described in recent publications [7,15]. A brief description of the main components of a pediatric FEES protocol is presented in Table 1. Modified procedures for breastfeeding [16] and examination of breastfeeding infants with laryngomalacia [17] have been described. Furthermore, protocols examining the safety and validity of FEES in neonatal intensive care units (NICUs) [5,18,19] have been published. 

## 3. Procedure Implementation

FEES implementation for infants (newborn to 12 months) depends upon several conditions, and special considerations apply. The child’s readiness to take nutritive trials is assessed. The decision to proceed with nutritive trials is based on the consistent presence of the oral reflexes (rooting, sucking, and swallowing) and the child’s ability to maintain an appropriate respiratory rate [20]. The ability of an infant to achieve and sustain a coordinated non-nutritive sucking pattern and demonstration of appropriate management of secretions are prerequisites to proceeding with any nutritive trials [7]. In most cases, additional monitoring use, such as pulse oximetry, is not required. If the child is on monitoring when the FEES examination takes place, the monitors are utilized. In oxygen-dependent and medically complex children, pulse oximetry should be used during the examination [7]. Adaptations are necessary for special populations, such as children in the cardiac unit, those that are medically fragile, those with a complex airway, and NICU infants. Nutritive trials start with a controlled presentation of minimal volumes of breast milk or formula. The infant’s ability to sustain a coordinated feeding pattern will dictate the decision for further presentation of nutritive trials. If the infant demonstrates sucking and swallowing competence, boluses of gradually increasing volumes are offered.

Another consideration is the child’s ability to reach and sustain an alert and quiet state for the nutritive trials. Research has shown an overall favorable completion rate of pediatric FEES [7,9]. During the procedure, children experience discomfort, which may result in crying, rendering cooperation much more difficult. Further improvements in participation and FEES completion can be based on understanding of the factors associated with failure to cooperate. Excessive crying is identified as the main barrier to participation [9]. In order to calm and soothe infants, some teams adopt special measures [18], whereas other teams report that most children cooperate with the procedure. The calming techniques employed include using sucrose solution, non-nutritive sucking, or breastfeeding [21]. Haller et al. reported that in some cases of “excessive crying”, the children were able to calm down for long enough to obtain a result from the FEES procedure, but in other cases, they would not calm at all [9]. Miller et al. reported that visual distraction strategies (including video clips and distraction toys) were beneficial during FEES [7]. Medical play therapy can help reduce anxiety and enhance the cooperation of children during medical procedures. Other reported causes of noncooperation are a nosebleed during FEES, vomiting, and refusal to latch for breastfeeding with the endoscope in place [9].

The consistencies used and the volumes of food or liquids tried widely vary in children, depending on the developmental phase of swallowing, etiology of dysphagia, comorbidities, barriers identified, and the reason for FEES referral. Therefore, the duration of the procedure also considerably varies and can take up to 30 minutes. Due to the differences in anatomy between infants, children, and adolescents, endoscopes of various sizes are used (from 1.9 to 4.1 mm) [21]. The standard-size endoscopes (3.5–4.0 mm) can be used in most children and infants. A smaller endoscope (2.0–2.2 mm) is used in NICU FEES.

## 4. The Pediatric Dysphagia Teams

The care of a child challenged by feeding and swallowing difficulties is teamwork. Pediatric dysphagia is increasing in prevalence and constitutes a significant challenge for families, society, healthcare professionals, and healthcare systems, especially regarding human resources, expertise, health service organization, and health providers’ resources allocation. A multidisciplinary, multiprofessional dysphagia team includes pediatricians, pediatric neurologists, pediatric intensivists, otorhinolaryngologists, speech–language pathologists, phoniatricians, pediatric pulmonologists, pediatric surgeons, pediatric gastroenterologists, nutrition specialists, and rehabilitation specialists, who provide coordinated and integrated care with continuity through care transitions. Within the pediatric dysphagia team, the allocation and distribution of tasks are varied and dynamic, determined by different professional developments of medical and healthcare disciplines in different countries and the relevant legal regulations [7,8,22]. A collaborative approach to the FEES examination by an otolaryngologist and a speech–language pathologist facilitates the examination and fosters best practices in managing pediatric dysphagia. Due to varying national practices and legislation, it is difficult to define generally accepted recommendations [22]. FEES training opportunities are widely available. In the USA, the American Speech Language and Hearing Association (ASHA) described a set of recommended FEES competencies for speech–language pathologists (SLPs). Especially in the case of medically fragile NICU infants, competency development and training are necessary to establish a multidisciplinary FEES practice [18]. In many European countries FEES is mainly performed by phoniatricians, for whom dysphagia management is part of their core competence [22]. Implementing FEES in pediatric dysphagia management requires knowledge of the developmental aspects of feeding, anatomic and physiological changes, and swallowing impairments associated with structural, neurologic, cardiorespiratory, or metabolic conditions.

## 5. Perspectives

Although pediatric FEES has evolved into a standard in the diagnosis of swallowing disorders, examination protocols, rating scales, and interpretation for children mostly lack validation [21]. A recent systematic review of studies reporting on protocols for pediatric FEES identified 22 studies reporting on FEES in 1547 infants, children, and adolescents with varying diagnoses [21]. Some studies indicated that the boluses examined were developmentally appropriate, whereas many studies did not describe the boluses used. The reporting of secretion pooling, laryngeal sensation, premature spillage and swallowing onset, penetration, aspiration, and residue are not consistent in pediatric FEES studies. No validated scale was used, with the exception of the penetration–aspiration scale used in one study. Furthermore, even the boundary between normal and abnormal spillage must be clarified in children [7]. 

## 6. Conclusions

FEES is a part of the clinical protocol for evaluating and managing dysphagia and is used in children of all ages. It provides visualization of the swallowing structures and their function and evaluation of the safety, efficiency, and adequacy of oral food and liquid intake. Future perspectives include standardized training in clinical FEES protocols to ensure the clinical competency of the pediatric FESS team members and the development and validation of standardized examination and interpretation protocols for pediatric FEES.

## Figures and Tables

**Table 1:** A pediatric FEES protocol.

**Preprocedure preparation**
History
Reason for referral
Current feeding status/difficulties
Diagnoses
Education of the family about the procedure
Oral sensorimotor skills
Level of alertness
Posture and position
Control of oral secretions
**Procedure preparation**
Nasal decongestion/anesthesia
Positioning
Food and utensils according to the developmental level and the reported usual method of intake
**Anatomy and physiology visualization**
Nasopharynx: the adequacy of velopharyngeal closure
Oropharynx
Hypopharynx and larynx at rest
Vocal cord mobility (abduction/adduction assessed as the child cries, phonates, coughs, or holds breath)
Pharyngeal squeeze
Secretion management and swallow frequency
Response to aspiration of secretions
Vocal quality (normal/wet)
Sensation
**Swallowing assessment: liquids, purees, solids**
Assessment of swallowing as the child drinks and eats various bolus consistencies
Swallowing onset time
Timely onset/delay in onset: Initiation of swallow when bolus head in valleculae/
in pyriform/no appreciable swallow initiation
Laryngeal penetration
Inconsistent, consistent, location
Aspiration
Prior to swallow, following swallow
Pharyngeal Residue
Location
Required multiple swallows to clear (spontaneous or at verbal cue)
**Compensatory and adaptive treatments**
Positioning
Rate of intake
Postural maneuvers
Alternating solids/liquids to clear pharyngeal residue
Effortful swallow

## Data Availability

Not applicable.
